# Remimazolam-Induced Anaphylaxis After Spinal Anesthesia: A Case Report and Literature Review †

**DOI:** 10.3390/jcm15114099

**Published:** 2026-05-26

**Authors:** Yumin Jo, Juhyun Kim, Sanghun Lee, Chaeseong Lim

**Affiliations:** 1 Department of Anesthesiology and Pain Medicine, Chungnam National University Hospital, Chungnam National University College of Medicine, Daejeon 35015, Republic of Korea; lemonny87@cnuh.co.kr (Y.J.); 02julia@cnuh.co.kr (J.K.); t10222@naver.com (S.L.); 2 Integrated Disease Research Institute, Chungnam National University College of Medicine, Daejeon 35015, Republic of Korea

**Keywords:** remimazolam, anaphylaxis, spinal anesthesia, tryptase, ECMO

## Abstract

Perioperative anaphylaxis, though rare, is a potentially life-threatening complication. While antibiotics and neuromuscular blocking agents are common triggers, benzodiazepine-induced reactions have been considered uncommon. Remimazolam, a novel benzodiazepine sedative, has gained widespread use in Korea due to its rapid onset, short recovery, hemodynamic stability, and availability of flumazenil. However, increasing utilization has coincided with rising reports of hypersensitivity. We report the case of a 62-year-old female undergoing contralateral total knee replacement under spinal anesthesia. Continuous remimazolam infusion was initiated, but within ten minutes the patient developed chest discomfort followed by abrupt hypotension and oxygen desaturation, requiring urgent conversion to general anesthesia. Following a remimazolam bolus and rocuronium administration, sudden cardiac arrest occurred. Return of spontaneous circulation (ROSC) was achieved after approximately 28 min of cardiopulmonary resuscitation with a cumulative intravenous epinephrine dose of approximately 17 mg, and veno-arterial extracorporeal membrane oxygenation (ECMO) was required. Post-ROSC transesophageal echocardiography demonstrated a transient anteroseptal regional wall motion abnormality; subsequent coronary angiography demonstrated no significant coronary disease, and computed tomography pulmonary angiography was negative for embolism, leaving acute hypersensitivity as the most plausible mechanism. Acute serum tryptase was elevated at 11.6 µg/L and normalized to 3.4 µg/L (the patient’s individual baseline) prior to discharge, satisfying the World Allergy Organization (WAO) criterion. A skin prick test performed four weeks later was positive for remimazolam and negative for rocuronium and the other coadministered agents. An expanded multi-database literature review identified 16 prior cases of remimazolam-induced anaphylaxis. Most described cardiovascular collapse as the predominant manifestation. To our knowledge, based on available literature, this is among the first reports of remimazolam-induced anaphylaxis occurring in the setting of high spinal anesthesia with sympathetic blockade. Vigilance and adherence to established anaphylaxis management guidelines are essential.

## 1. Introduction

Perioperative anaphylaxis, although rare, remains a potentially life-threatening complication that no anesthesiologist can ever afford to underestimate when encountering it. The most common pharmacologic triggers include antibiotics and neuromuscular blocking agents, while contrast media and latex used during surgery also warrant careful consideration.

The incidence of benzodiazepine-induced anaphylaxis has historically been considered very uncommon [1]. Remimazolam, an ultra-short-acting benzodiazepine sedative, has gained widespread use in Korea since its introduction. Owing to its rapid onset and swift recovery profile, it is frequently employed for continuous infusion sedation and as an alternative to propofol in total intravenous anesthesia. Its favorable hemodynamic stability and the availability of a specific reversal agent, flumazenil, further enhance its clinical utility.

However, within the past year, two cases of remimazolam-induced anaphylaxis were observed at the authors’ institution, prompting a review of the relevant literature. One patient consented to publication, and we herein present that case with written informed consent obtained. In addition to reporting the clinical course, we aim to analyze the potential mechanisms underlying the reaction and to summarize pertinent findings from previous reports. This case highlights important safety considerations regarding the use of remimazolam. To our knowledge, based on available literature, this is among the first reports of remimazolam-induced anaphylaxis occurring in the context of high spinal anesthesia with sympathetic blockade.

## 2. Materials and Methods

We conducted a focused literature review of published cases and reviews of remimazolam-induced anaphylaxis, with the search re-executed on 9 May 2026 to incorporate recent literature. The original search strategy was substantially expanded after peer review.

Databases searched: PubMed/MEDLINE, Embase, Cochrane Library, Web of Science, and Google Scholar (the last to capture grey literature and non-MEDLINE-indexed sources).

Search string: (“remimazolam”[All Fields]) AND (“anaphylaxis” OR “hypersensitivity” OR “allergy” OR “allergic reaction”). The broader terms were chosen to capture reports indexed only under “hypersensitivity” or “allergy” rather than “anaphylaxis”.

Inclusion criteria: (i) original case reports, case series, or systematic reviews describing remimazolam-induced anaphylaxis or hypersensitivity reactions meeting World Allergy Organization (WAO) clinical criteria; (ii) full text available; (iii) English-language publications (with one Japanese-language case translated for inclusion after identification through reference cross-checking).

Exclusion criteria: conference abstracts without full text, animal studies, pharmacovigilance database analyses (cited in Discussion but not pooled in Table 1), and reports of remimazolam use without hypersensitivity events.

**Figure 1:**
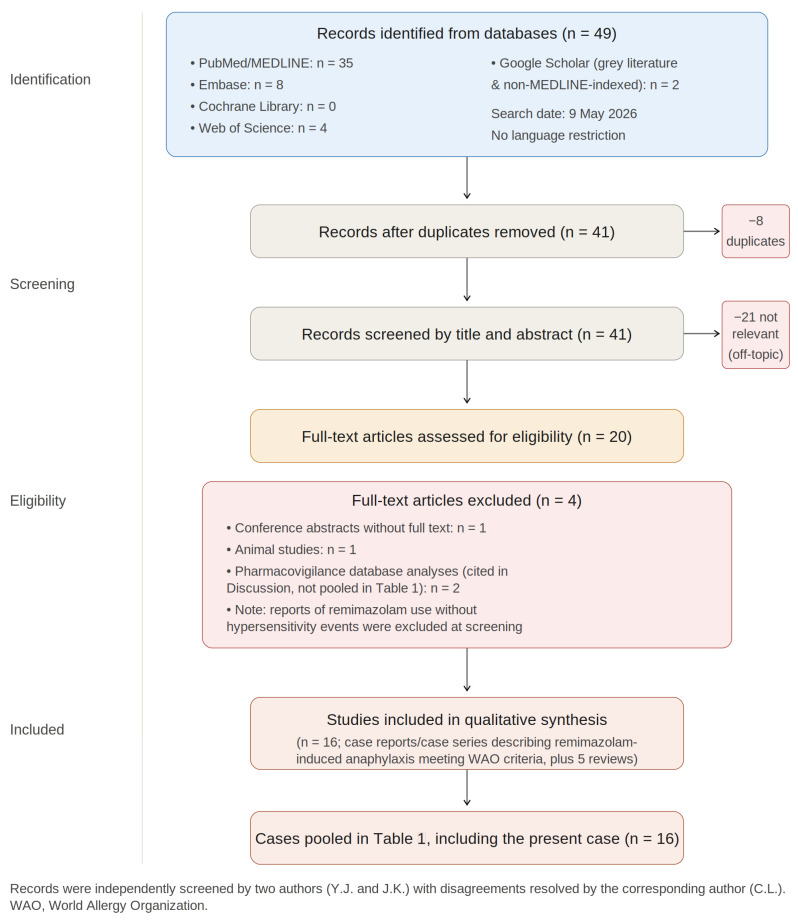
PRISMA-style flow of records for the literature review of remimazolam-induced anaphylaxis. The search was executed on 9 May 2026 across PubMed/MEDLINE, Embase, Cochrane Library, Web of Science, and Google Scholar. Records were independently screened by two authors (Y.J. and J.K.), with disagreements resolved by the corresponding author (C.L.). Sixteen case reports/series meeting World Allergy Organization (WAO) clinical criteria for anaphylaxis were pooled in Table 1 (including the present case); five reviews were retained for narrative synthesis in the Discussion. WAO, World Allergy Organization.

Records were independently screened by two authors (Y.J. and J.K.), with disagreements resolved by the corresponding author (C.L.). The PRISMA-style flow of records is presented in Figure 1.

Compared with the original single-database, single-term strategy (“remimazolam and anaphylaxis” in PubMed), the expanded multi-database, broader-term strategy captured additional reports indexed under “hypersensitivity” or “allergic reaction” (e.g., Blake et al. [2]) and reports published in journals not indexed in MEDLINE (e.g., Kwon et al. [3]), as well as relevant pharmacovigilance signal-detection studies ([4,5]). All retrieved articles meeting inclusion criteria were thoroughly examined and compared with the present case.

**Table 1:** Summary of reported cases of remimazolam-induced anaphylaxis (including the present case).

Author/Year	Age/Sex	Dose (mg)	Anesthesia Type	Symptoms/Signs	Tryptase/Skin Test	Cardiac Arrest	Confirmation Method	Additional Findings/Comments
Uchida 2022 [6] (case 1)	74/M	4	GA induction	Hypotension, desaturation	+/NA	No	SPT not performed	Sensitization suspected
Uchida 2022 [6] (case 2)	59/M	9	GA induction	Discomfort, hypotension	+/−	No	SPT negative; tryptase rise	—
Yamaoka 2022 [7]	78/M	>12	GA induction	Hypotension, desaturation, high airway pressure	+/+	No	Tryptase + SPT positive	—
Hasushita 2022 [8]	72/M	~72	GA induction	Cardiac arrest, skin erythema	+/+	Yes (ROSC 6 min)	Tryptase + SPT positive	Prior midazolam exposure tolerated; allergic to acemetacin, kikyo-sekko
Hu 2023 [9]	41/M	10	Procedural sedation (colonoscopy)	Stridor, erythema, lip swelling	NA/−	No	Clinical/temporal	Onset within 1 min
Tsurumi 2021 [10]	32/M	12	GA induction	Hypotension, desaturation, facial flushing	−/+	No	SPT positive	Cross-reactivity with midazolam suspected
Kim KM 2022 [11] (case 1)	65/M	98.8	GA induction	Hypotension, ST change	+/−	No	Tryptase rise	—
Kim KM 2022 [11] (case 2)	69/M	78	GA induction	Hypotension, ST change	+/NA	No	Tryptase rise	—
Kim KM 2022 [11] (case 3)	66/M	57.4	GA induction	Cardiac arrest	+/−	Yes (ROSC 5 min)	Tryptase rise	Refractory to repeated 200 µg epinephrine
Kim KM 2022 [11] (case 4)	23/F	26	GA induction	Rash, cough, chest tightness	NA/−	No	Clinical	Crohn’s disease
Kim KM 2022 [11] (case 5)	33/F	8.4	GA induction	Rash, dyspnea	+/−	No	Tryptase rise	—
Nakai 2024 [12]	75/M	8	GA induction	Cardiac arrest, bronchospasm	+/NA	Yes (ROSC 2 min)	Skin test for other drugs negative	Prior brotizolam tolerated; sensitization to midazolam/brotizolam considered
Lee S 2023 [13]	51/F	10.4	GA induction	Skin rash, hypotension	−/−	No	Positive provocation (1 mg)	Provocation test positive
Mani 2024 [14]	77/M	2.5	GA induction	Hypotension, desaturation	+/NA	No	Tryptase rise	Mastocytosis; multiple drug allergies
Kwon 2026 [3]	68/M	14	GA induction	Bronchospasm, hypotension, transient AV block (Kounis-like)	NA/NA	No (peri-arrest)	Clinical/exclusion; patient declined allergy work-up	Type I Kounis syndrome features
Blake 2026 [2]	14/[M/F]	NA	Procedural sedation	Localized urticarial reaction at IV site	NA/NA	No	Clinical/temporal	Pediatric case; mild localized hypersensitivity
Present case	62/F	>13	Spinal anesthesia + GA conversion	Chest discomfort, refractory hypotension, cardiac arrest (PEA/asystole with intermittent V.fib)	+/+	Yes (ROSC 28 min, total epinephrine ~17 mg, ECMO required)	Tryptase + SPT positive; CAG normal; CTPA negative	Transient anteroseptal RWMA on TEE with normal CAG (compatible with Kounis-type allergic coronary vasospasm or anaphylactic myocardial stunning); re-exposure 2 weeks prior; high spinal block with head-down positioning as aggravating factor

M, male; F, female. Tryptase: + = elevated above the individual baseline meeting WAO criterion (peak ≥ 1.2 × baseline + 2 µg/L); − = within normal range; NA = not measured or not performed. Skin test (SPT/IDT): + = positive (wheal ≥ 3 mm above negative control); − = negative; NA = not performed or not available. GA, general anesthesia; ROSC, return of spontaneous circulation; AV, atrioventricular; ECMO, extracorporeal membrane oxygenation; SPT, skin prick test; IDT, intradermal test; CAG, coronary angiography; CTPA, CT pulmonary angiography; PEA, pulseless electrical activity; RWMA, regional wall motion abnormality; TEE, transesophageal echocardiography; WAO, World Allergy Organization.

AI-assisted manuscript preparation: During the preparation of this manuscript, we employed an artificial intelligence language model (Claude, version 3.5) to support non-analytical tasks. The tool was used to assist in:

(i) improving clarity and readability of draft text during revision,
(ii) generating schematic figures and illustrative diagrams based on author-provided concepts,
(iii) organizing and re-ordering reference citations according to journal formatting requirements

All outputs generated by the AI tool were critically reviewed, edited, and validated by the authors to ensure accuracy, originality, and compliance with scientific standards. The final responsibility for the content rests entirely with the authors.

## 3. Case Presentation

### 3.1. Patient Background and Anesthetic Course

A 62-year-old female (height 163 cm, weight 76 kg, body mass index 28.6 kg/m^2^; American Society of Anesthesiologists physical status II) had undergone total knee replacement two weeks earlier under spinal anesthesia, during which the procedure was successfully completed with continuous remimazolam infusion for sedation. Her medical history included hypertension and diabetes mellitus, both under outpatient pharmacologic management; the patient continued her usual self-administered medications throughout the perioperative period in accordance with institutional policy, although the specific agents were not documented in the chart. She had no known drug allergies and no other comorbidities. Preoperative evaluation showed normal pulmonary function tests, and transthoracic echocardiography demonstrated a left ventricular ejection fraction of 60% with normal contractility, borderline concentric left ventricular hypertrophy, and left atrial enlargement—findings compatible with chronic hypertensive remodeling. The previous spinal anesthesia for the first-side total knee replacement, performed with the same protocol two weeks earlier, had been entirely uneventful. Consequently, the anesthetic plan for the contralateral knee surgery was designed to follow the same protocol.

Spinal anesthesia was performed under ultrasound guidance with the patient in the lateral decubitus position, at the L5–S1 interspace, using a 25-gauge Quincke spinal needle following skin infiltration with 2% lidocaine. A total of 0.5% heavy bupivacaine 12 mg combined with morphine 0.1 mg was administered intrathecally after confirmation of free cerebrospinal fluid (CSF) flow. After spinal injection, the patient was repositioned and placed transiently in the head-down (Trendelenburg) position to facilitate placement of a left brachial arterial line and a 16-gauge peripheral intravenous catheter in the right external jugular vein. The head-down maneuver, while routine at our institution for vascular access, is a recognized factor that can promote cephalad migration of hyperbaric local anesthetic and may have contributed to a higher-than-expected block height in this patient. Standard monitoring included pulse oximetry (SpO_2_), noninvasive blood pressure (NIBP), electrocardiography (EKG), pulse variability index (PVi), continuous hemoglobin (SpHb), and oxygen reserve index (ORi). Oxygen was supplied at 5 L/min via face mask with end-tidal CO_2_ monitoring.

**Figure 2:**
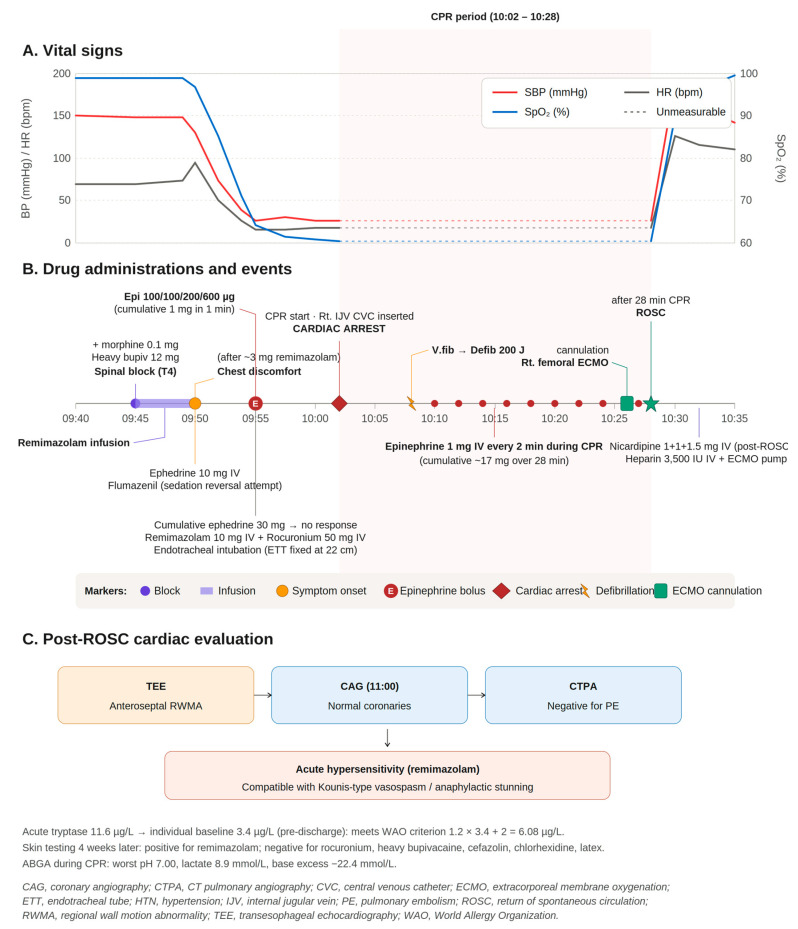
Clinical timeline of remimazolam-induced anaphylaxis. (**A**) Trends in systolic blood pressure (SBP, red), heart rate (HR, grey), and peripheral oxygen saturation (SpO_2_, blue) from 09:40 to 10:35. Dashed segments indicate the period during cardiopulmonary resuscitation (CPR) in which the parameters were not measurable. (**B**) Drug-administration timeline and key events: spinal block at 09:45 with sensory level T4 (heavy bupivacaine 12 mg + morphine 0.1 mg); chest discomfort at 09:50 after approximately 3 mg of remimazolam infusion, ephedrine 10 mg IV and flumazenil administered on a working diagnosis of high spinal block; escalating intravenous epinephrine boluses (100, 100, 200, and 600 µg; cumulative 1 mg) following cumulative ephedrine 30 mg without response, with induction (remimazolam 10 mg IV + rocuronium 50 mg IV) and tracheal intubation at 09:55; cardiac arrest at 10:02 with chest compressions and right internal jugular central venous catheter placement; biphasic defibrillation 200 J for transient ventricular fibrillation at 10:08; intravenous epinephrine 1 mg every 2 min through CPR (cumulative dose ~17 mg over 28 min); right femoral veno-arterial extracorporeal membrane oxygenation (ECMO) cannulation at 10:26; return of spontaneous circulation (ROSC) at 10:28; post-ROSC hypertension managed with nicardipine (1 + 1 + 1.5 mg IV); heparinization (3500 IU IV) and ECMO pump initiation at 10:32. (**C**) Post-ROSC cardiac evaluation: transesophageal echocardiography (TEE) demonstrated transient anteroseptal regional wall motion abnormality (RWMA); coronary angiography (CAG) at 11:00 was normal; computed tomography pulmonary angiography (CTPA) was negative for pulmonary embolism. The constellation of findings was compatible with allergic coronary vasospasm (Kounis syndrome type I) or anaphylaxis-induced myocardial stunning. The acute tryptase value of 11.6 µg/L was elevated relative to the individual baseline of 3.4 µg/L drawn pre-discharge (WAO threshold = 1.2 × 3.4 + 2 = 6.08 µg/L). CAG, coronary angiography; CTPA, CT pulmonary angiography; CVC, central venous catheter; ECMO, extracorporeal membrane oxygenation; ETT, endotracheal tube; HTN, hypertension; IJV, internal jugular vein; PE, pulmonary embolism; ROSC, return of spontaneous circulation; RWMA, regional wall motion abnormality; TEE, transesophageal echocardiography; WAO, World Allergy Organization.

The clinical timeline of the event, taken directly from the anesthesia record, is summarized below. The cardiopulmonary resuscitation (CPR) drug-administration sequence is presented in detail in Figure 2.

09:16: Patient arrived in the operating room. Standard monitoring established.

09:20: Left brachial arterial line placed; 16-gauge IV catheter inserted into the right external jugular vein during transient head-down positioning.

09:45: Spinal block performed as above; sensory level confirmed at T4. Continuous remimazolam infusion initiated for sedation at approximately 0.5 mg/kg/h.

09:50: After approximately 3 mg of remimazolam had been infused as a loading dose, the patient reported chest discomfort and developed hypotension. The sensory level was rechecked and remained at T4. Crystalloid loading was initiated and ephedrine 10 mg IV was administered. The remimazolam infusion was stopped and flumazenil was given in an attempt to reverse sedation, on the working assumption that symptoms reflected excessive sedative effect superimposed on a high spinal block.

09:55: Abrupt deterioration with profound hypotension and falling SpO_2_. Additional ephedrine 10 mg boluses were given to a cumulative dose of 30 mg without hemodynamic response. The induction sequence began with remifentanil continuous infusion, remimazolam 10 mg IV, and rocuronium 50 mg IV; epinephrine was administered in escalating IV boluses (100 µg, 100 µg, 200 µg, 600 µg; cumulative 1 mg during this minute) as decompensation accelerated. Endotracheal intubation was performed; capnography and bilateral breath sounds were confirmed, and the tube was fixed at 22 cm.

10:02: Cardiac arrest declared. Chest compressions commenced; right internal jugular central venous catheter placed during ongoing CPR.

10:08: A brief ventricular fibrillation episode was identified and treated with biphasic 200 J defibrillation; advanced cardiac life support otherwise managed predominantly pulseless electrical activity and asystole.

10:10–10:24: Ongoing CPR. Intravenous epinephrine 1 mg was administered every 2 min. Norepinephrine continuous infusion was started.

10:26: Epinephrine 1 mg IV; right femoral veno-arterial ECMO cannulation initiated by the cardiothoracic surgery team.

10:28: ROSC achieved after approximately 28 min of CPR. Cumulative IV epinephrine dose was approximately 17 mg.

10:29: Post-ROSC hypertensive response treated with nicardipine 1 mg + 1 mg + 1.5 mg IV (total 3.5 mg).

10:32: Heparin 3500 IU IV was given and ECMO pump flow was established.

Post-ROSC: Transesophageal echocardiography (TEE) demonstrated a regional wall motion abnormality (RWMA) in the anteroseptal wall. The patient was transferred to the cardiac catheterization laboratory under ambu-bag ventilation and ECMO support.

11:00: Coronary angiography (CAG) demonstrated no significant coronary artery disease. Computed tomography pulmonary angiography (CTPA) was negative for pulmonary embolism.

Subsequent: The patient was transferred to the surgical intensive care unit on ECMO and portable ventilation. Dexamethasone 5 mg and pheniramine 4 mg were administered intravenously as adjunctive anti-allergic therapy after initial resuscitation. Serum tryptase was drawn during the acute phase. Serial arterial blood gas analyses during the resuscitation documented progressive lactic acidosis (worst pH 7.00, lactate 8.9 mmol/L, base excess −22.4 mmol/L), which resolved over the subsequent hours on ECMO support.

### 3.2. Outcome and Confirmatory Work-Up

The patient was successfully weaned from ECMO one day after initiation and was discharged without sequelae one week later. She has since continued follow-up at the orthopedic outpatient clinic, where she reports severe knee pain and is currently requesting surgery on the operated knee.

Acute serum tryptase, drawn during the event, was elevated at 11.6 µg/L; a repeat measurement prior to discharge, after recovery of the mast-cell compartment, had returned to 3.4 µg/L (this convalescent value, drawn well beyond the post-anaphylaxis window in which tryptase remains elevated, represents the patient’s individual baseline). Application of the World Allergy Organization (WAO) consensus formula (peak ≥ 1.2 × baseline + 2 µg/L) yields a threshold of 1.2 × 3.4 + 2 = 6.08 µg/L, which the acute value of 11.6 µg/L clearly exceeds. The clinical event was classified as Ring and Messmer Grade IV (cardiac arrest) on the basis of the documented cardiac arrest with cardiopulmonary resuscitation required for ROSC.

We note that the absolute peak value (11.6 µg/L) is only moderately elevated despite the severity of the clinical event. Possible explanations include dilution from large-volume crystalloid resuscitation, absence of overt cutaneous mediator release, and a potential contribution of non-IgE-mediated pathways alongside an IgE-mediated component. The formal WAO criterion is nonetheless met, and the diagnosis of anaphylaxis is further supported by the positive skin prick test for remimazolam (see below) and the clinical course.

Allergy work-up. Four weeks after the event—i.e., outside the recognized refractory period for skin testing—the patient was evaluated at the allergy clinic. Skin prick testing (SPT) followed by intradermal testing (IDT) was performed for the following panel of agents administered during the index anesthetic (Table 2):

Interpretation. Skin testing was positive only for remimazolam and negative for all other tested agents, including rocuronium. Combined with the elevated acute serum tryptase, its normalization at recovery, and the temporal sequence of events (onset of decompensation shortly after the remimazolam bolus and before any clinical effect of rocuronium would be expected to influence hemodynamics), these results supported a diagnosis of remimazolam-induced anaphylaxis and excluded rocuronium as the culprit agent.

## 4. Discussion

Anaphylaxis induced by benzodiazepines has been considered exceedingly rare. However, according to Kim et al. [11], remimazolam has demonstrated a relatively higher incidence of approximately 0.18%. Since its introduction in Korea, the use of remimazolam has steadily increased, accompanied by multiple reports of anaphylaxis and similar adverse reactions. Traditionally, NMBAs such as rocuronium, antibiotics, and, less frequently, opioids including fentanyl or remifentanil have been implicated as causative agents. Nevertheless, growing concern has emerged among clinicians regarding the potential risk associated with remimazolam, supported by recent pharmacovigilance signal-detection studies of the FDA Adverse Event Reporting System (FAERS) and the Japanese Adverse Drug Event Report (JADER) databases [4,5,15], which identify anaphylaxis and laryngeal edema as significant serious adverse-event signals.

In addition to IgE-mediated anaphylaxis directly attributable to remimazolam, reports have suggested that dextran 40, an excipient in the formulation, may provoke non-IgE-mediated hypersensitivity reactions (historically termed “anaphylactoid reactions”). This has prompted ongoing discussion about the underlying mechanisms of remimazolam-related hypersensitivity.

The classical form of IgE-mediated anaphylaxis is antibody-dependent. In sensitized patients with prior exposure to the same antigen, mast cells and basophils undergo degranulation, releasing chemical mediators such as histamine and tryptase. This cascade results in vasodilation, bronchoconstriction, and hypotension, producing severe clinical manifestations. Importantly, such reactions can occur even with minimal re-exposure to the antigen, regardless of the dose administered.

In contrast, non-IgE-mediated hypersensitivity reactions occur without antibody involvement, typically through direct mast cell or basophil activation or complement activation (as with dextran-induced reactions). The clinical manifestations are indistinguishable from those of IgE-mediated anaphylaxis. Sensitization is not required, and such reactions may exhibit dose-dependent characteristics.

Because of these mechanistic differences, non-IgE-mediated hypersensitivity reactions often present with negative skin test results and may not demonstrate elevated serum tryptase levels, making definitive diagnosis challenging. Nevertheless, both IgE-mediated and non-IgE-mediated reactions manifest as systemic hypersensitivity events characterized by abrupt hemodynamic instability, respiratory compromise, and (often) cutaneous findings. In either case, immediate administration of epinephrine remains the cornerstone of treatment.

### 4.1. Treatment Principles Aligned with Current Guidelines

Current EAACI Anaphylaxis Guidelines [16] and the international consensus on suspected immediate perioperative allergic reactions [17] uniformly identify intramuscular or intravenous epinephrine as the first-line, time-critical intervention in anaphylaxis. Adjunctive measures include high-flow oxygen, large-volume crystalloid resuscitation, airway management, and additional vasopressors (norepinephrine, vasopressin) when refractory hypotension persists. Antihistamines (H_1_- and H_2_-blockers) and corticosteroids are adjunctive only; they do not reverse the underlying mast-cell-mediated cascade and must never delay epinephrine administration.

In our patient, the institutional perioperative anaphylaxis protocol was followed. Ephedrine 10 mg IV was administered first when chest discomfort and hypotension developed, on the working assumption of a high spinal block. When successive ephedrine boluses to a cumulative dose of 30 mg failed to restore blood pressure, intravenous epinephrine was begun as escalating boluses (100 µg, 100 µg, 200 µg, and 600 µg within the first minute, totaling 1 mg) and tracheal intubation was performed using remifentanil, remimazolam 10 mg IV, and rocuronium 50 mg IV. Cardiac arrest occurred shortly after intubation; advanced cardiac life support followed standard ACLS protocols, with closed-chest compressions, 1 mg IV epinephrine boluses every 2 min, biphasic defibrillation at 200 J when a ventricular fibrillation episode was identified at 10:08, and a norepinephrine continuous infusion. Veno-arterial ECMO was cannulated via the right femoral vessels during ongoing CPR. ROSC was achieved after approximately 28 min of CPR with a total cumulative IV epinephrine dose of approximately 17 mg. Post-ROSC hypertension required nicardipine titration, and the patient was systemically heparinized for ECMO. Dexamethasone 5 mg and pheniramine 4 mg were administered intravenously after the initial resuscitation, consistent with their role as adjuncts rather than first-line agents.

### 4.2. The Flumazenil Pitfall in Suspected High Spinal Block

Our case illustrates this point as a clinical pitfall. When the patient first reported chest discomfort during remimazolam infusion in the setting of a high spinal block, the clinical team initially interpreted the symptoms as an excessive sedative effect superimposed on sympathetic blockade and administered flumazenil to reverse sedation. Hemodynamic deterioration nevertheless progressed rapidly, and the true mechanism—acute hypersensitivity—became apparent only retrospectively. Clinicians should be aware that an attempt at benzodiazepine reversal does not modify the underlying allergic cascade and should not delay administration of epinephrine, which remains the only proven first-line therapy.

### 4.3. Cardiovascular Collapse in Remimazolam Anaphylaxis: Comparison with the Literature

Taken together, in the previously reported cases of cardiac arrest induced during general anesthesia, ROSC was achieved within six minutes of chest compression and with cumulative epinephrine doses not exceeding 2 mg. In contrast, the present case required approximately 28 min of chest compression, a cumulative IV epinephrine dose of approximately 17 mg, and ECMO support to achieve ROSC. The expanded case-level summary is presented in Table 1.

More recently, the expanded literature search identified an additional Korean case: Kwon et al. [3] reported a 68-year-old man who developed life-threatening anaphylaxis with profound hypotension, refractory bronchospasm, and transient atrioventricular block consistent with type I Kounis syndrome following a 14 mg remimazolam induction. Hemodynamic recovery was achieved with an IV epinephrine bolus and infusion. This case is notable for two reasons: (i) it illustrates rare cardiac conduction manifestations of remimazolam anaphylaxis, and (ii) it was published in a journal not indexed in MEDLINE, underscoring the importance of multi-database searching for emerging adverse-event literature.

### 4.4. Severity, Sensitization, and the Role of High Spinal Anesthesia as an Aggravating Factor

Several factors likely contributed to the catastrophic severity of the present case. First, severe anaphylaxis was supported by an elevated acute serum tryptase (11.6 µg/L) and a positive skin prick test for remimazolam (see Section 3.2). The diagnostic criteria for anaphylaxis according to the WAO consensus were therefore met, with a Ring and Messmer severity grade of IV (cardiac arrest).

Second, the patient had a recent prior exposure to remimazolam two weeks earlier under the same protocol without adverse events. This temporal pattern is consistent with an IgE-mediated mechanism: the initial exposure may have sensitized the patient, and the subsequent re-exposure precipitated a pronounced anaphylactic reaction [18]. The short interval between exposures may have intensified the response. This sequence is more consistent with an IgE-mediated reaction to remimazolam itself than with a non-IgE-mediated hypersensitivity reaction attributable to dextran 40, although both pathways may contribute additively.

Third, and uniquely to our case, a high spinal block with sympathetic blockade was a major aggravating context for the anaphylactic event. Spinal anesthesia of any height produces a degree of sympatholysis with arteriolar and venous vasodilation, baroreceptor reflex blunting, and reduced cardiac preload. When the block extends to a T4 level (as in our patient, confirmed twice on chart review), thoracic cardiac accelerator fibers are inhibited and the compensatory reserve is markedly diminished. The unexpectedly high block height after intrathecal 12 mg of 0.5% heavy bupivacaine at L5–S1 in the lateral decubitus position is plausibly attributable to the transient head-down (Trendelenburg) position adopted immediately after spinal injection to facilitate vascular access. In this hemodynamic substrate, the additional vasodilatory and capillary-leak effects of anaphylaxis precipitate a deeper and more refractory hypotension than would occur in an unblockade patient—explaining, at least in part, the failure of escalating ephedrine and the requirement for very large cumulative doses of epinephrine and ECMO.

### 4.5. Distinctive Cardiac Findings: Anaphylactic Stunning or Kounis Syndrome?

A distinctive feature of the present case was the post-ROSC finding of transient anteroseptal regional wall motion abnormality (RWMA) on transesophageal echocardiography in conjunction with a coronary angiogram that demonstrated no significant coronary artery disease and a negative computed tomography pulmonary angiogram. Importantly, the preoperative transthoracic echocardiogram had documented normal left ventricular contractility (ejection fraction 60%) with no segmental wall-motion abnormality, providing a critical baseline reference. The transient anteroseptal RWMA observed after ROSC therefore represents a new abnormality temporally associated with the anaphylactic event, rather than a pre-existing wall-motion defect. Acute myocardial infarction and pulmonary thromboembolism—the leading differential diagnoses for cardiac arrest with regional myocardial dysfunction—were therefore excluded. The remaining plausible explanations are anaphylaxis-induced myocardial stunning and allergic coronary vasospasm consistent with type I Kounis syndrome [19]. The recent report by Kwon et al. [3] described a comparable cardiac presentation—transient atrioventricular conduction block in association with remimazolam-induced anaphylaxis in a patient without underlying coronary disease—and the authors proposed type I Kounis syndrome as the most consistent mechanism. While we cannot distinguish definitively between mast-cell-mediator-driven myocardial stunning and transient allergic coronary vasospasm in our patient, the constellation of findings is compatible with the latter. To our knowledge, this is among the first reports of remimazolam-induced anaphylaxis in which transient RWMA was documented and coronary disease was prospectively excluded by angiography, contributing additional evidence that the cardiac manifestations of remimazolam hypersensitivity extend beyond simple vasodilatory hypotension.

### 4.6. Causality Assessment and Differential Diagnosis

Because the index event occurred against a backdrop of multiple coadministered drugs and concurrent high spinal anesthesia, we conducted a structured causality assessment using the Naranjo Adverse Drug Reaction Probability Scale [20] applied to remimazolam. The individual item scores and rationale are summarized in Table 3.

We also considered the differential diagnosis explicitly. The combination of hypotension and desaturation shortly after a documented T4 sensory block can resemble a high spinal block, and the absence of cutaneous manifestations complicates the diagnosis of anaphylaxis. Table 4 summarizes the discriminating features and how each applied to our patient.

We also note the absence of cutaneous signs (rash, urticaria, or angioedema) at the time of the event. Cutaneous manifestations are reported in approximately 50–80% of perioperative anaphylaxis cases but may be absent or unrecognized in 20–50%, particularly under the drapes during surgery or in patients receiving vasopressors that may mask flushing [17]. The absence of cutaneous signs does not exclude anaphylaxis and is well documented in remimazolam-induced cases, including several in Table 1 (e.g., Kim KM 2022 [11] cases 1–3).

### 4.7. Limitations

Several limitations should be acknowledged. First, as a single-patient observation, causal inferences about the relative contributions of high spinal anesthesia and anaphylaxis to the prolonged resuscitation cannot be drawn definitively. Second, although our allergy work-up was extensive, certain confirmatory tests (such as a graded intravenous provocation challenge with remimazolam, or measurement of serum specific IgE) were not performed because of the severity of the index reaction. Third, although the temporal pattern (prior tolerance two weeks earlier followed by severe reaction on re-exposure) favors an IgE-mediated mechanism, we could not formally distinguish IgE-mediated anaphylaxis to remimazolam itself from a dextran-40-related non-IgE-mediated hypersensitivity reaction; both pathways may contribute additively. Fourth, the expanded literature review, despite covering five databases, may still have missed reports in non-English-language journals or unindexed sources, and we therefore frame any priority claims as “to our knowledge, based on available literature.” Fifth, a formal risk-of-bias appraisal of each included report (e.g., using the JBI tool for case reports) was not applied, given that the goal of the review was descriptive synthesis rather than meta-analytic estimation.

This report adheres to the CARE (CAse REport) guidelines for case reporting; a completed CARE checklist is provided as Supplementary Material (Table S1).

## 5. Conclusions

Although uncommon, anaphylaxis associated with remimazolam should always be considered when unexplained hemodynamic instability develops shortly after its administration, and the increasing global use of this agent has been accompanied by a proportional rise in reported cases. Vigilance is therefore essential when administering remimazolam. Caution should be exercised in the setting of high spinal anesthesia, where hemodynamic instability may be aggravated should anaphylaxis occur. At the first sign of suspected hypersensitivity, remimazolam administration should be discontinued immediately and treatment initiated according to established guidelines, with intravenous epinephrine as the first-line intervention; antihistamines and corticosteroids serve only as adjuncts and should never delay epinephrine.

## Figures and Tables

**Table 2:** Allergy work-up performed four weeks after the index event. Skin prick testing (SPT) was followed by intradermal testing (IDT) for each agent. A wheal diameter ≥ 3 mm greater than the saline negative control was considered a positive response.

Agent	Concentration Tested	Result
Remimazolam	1 mg/mL undiluted (SPT); 0.01 mg/mL and 0.1 mg/mL (IDT)	Positive
Rocuronium	10 mg/mL undiluted (SPT); diluted 1:100 and 1:10 (IDT)	Negative
Heavy bupivacaine 0.5%	5 mg/mL undiluted (SPT); 0.5 mg/mL (IDT)	Negative
Morphine (intrathecal adjunct)	1 mg/mL (SPT); 0.1 mg/mL (IDT—non-specific histamine release; interpreted with caution)	Negative
Cefazolin	2 mg/mL (SPT); 0.02 mg/mL and 0.2 mg/mL (IDT)	Negative
Chlorhexidine	0.5 mg/mL (SPT); 0.002 mg/mL (IDT)	Negative
Latex extract	commercial extract per manufacturer (SPT)	Negative

SPT, skin prick test; IDT, intradermal test. Histamine (10 mg/mL) and 0.9% saline were included as positive and negative controls, respectively, with the expected reactions. A wheal diameter ≥ 3 mm greater than the saline negative control was considered a positive response. Morphine is known to cause non-specific cutaneous histamine release; its skin-test result was therefore interpreted with caution and was negative in the present case.

**Table 3:** Naranjo Adverse Drug Reaction Probability Scale applied to remimazolam in the present case. A total score of +8 corresponds to a probable adverse drug reaction.

Naranjo Item	Score	Rationale For The Present Case
1. Are there previous conclusive reports on this reaction?	+1	Multiple published case reports of remimazolam-induced anaphylaxis (e.g., [3,6,9,11,12,14]).
2. Did the adverse event appear after the suspected drug was administered?	+2	Symptoms began within minutes of remimazolam infusion and worsened after the IV bolus.
3. Did the adverse reaction improve when the drug was discontinued or a specific antagonist was administered?	+1	Hemodynamic recovery followed cessation of remimazolam and administration of intravenous epinephrine, the specific treatment for anaphylaxis.
4. Did the adverse reaction reappear when the drug was readministered?	0	No rechallenge attempted (contraindicated given the severity of the index event).
5. Are there alternative causes that could on their own have caused the reaction?	+1	Rocuronium and dextran-40 considered; rocuronium excluded by negative skin testing and timing. High spinal block excluded by failure to respond to ephedrine 30 mg and need for ~17 mg of epinephrine.
6. Did the reaction reappear when a placebo was given?	0	Not applicable.
7. Was the drug detected in blood or other fluids in toxic concentrations?	0	Not measured.
8. Was the reaction more severe when the dose was increased, or less severe when the dose was decreased?	+1	Severe deterioration followed the 10 mg IV bolus after the patient was already symptomatic during the loading infusion.
9. Did the patient have a similar reaction to the same or similar drugs in any previous exposure?	+1	IgE-mediated sensitization is suggested by uneventful tolerance two weeks earlier followed by severe reaction on re-exposure to the same agent.
10. Was the adverse event confirmed by any objective evidence?	+1	Elevated acute serum tryptase (11.6 µg/L; meeting the WAO 1.2× baseline + 2 µg/L threshold) and positive skin prick test for remimazolam at 4 weeks.
Total	+8	Probable (≥9 = definite, 5–8 = probable, 1–4 = possible, ≤0 = doubtful)

**Table 4:** Differential diagnosis: high spinal block versus anaphylaxis, with findings in the present case.

Feature	High Spinal Block	Anaphylaxis	Findings in the Present Case
Hypotension	Gradual, dose- and level-dependent; usually responsive to ephedrine and fluid	Abrupt, profound; refractory to fluids and large vasopressor doses	Refractory: ephedrine 30 mg failed; ~17 mg epinephrine required ⇒ favors anaphylaxis
Bradycardia	Common (Bezold-Jarisch reflex with T1–T4 sympatholysis)	May occur, but tachycardia more typical until pre-arrest	Heart rate trend not predominantly bradycardic ⇒ favors anaphylaxis
Respiratory pattern	Hypoventilation/apnea from phrenic involvement at very high levels (C3–C5); usually no bronchospasm	Bronchospasm, increased airway pressures, desaturation despite ventilation	SpO_2_ fell despite preoxygenation and assisted ventilation ⇒ favors anaphylaxis
Cutaneous signs	Absent	Present in ~50–80% of cases (but absent in 20–50%)	Absent—does not rule out anaphylaxis (well-recognized)
Serum tryptase	Not elevated	Elevated (meeting WAO criterion)	11.6 µg/L vs. 3.4 µg/L baseline = positive WAO criterion ⇒ supports anaphylaxis
Skin testing (delayed)	Negative	Positive for culprit agent	Positive for remimazolam; negative for rocuronium and others ⇒ supports anaphylaxis
Response to epinephrine	Minimal (block not reversible by epinephrine)	Hemodynamic recovery with epinephrine	BP and SpO_2_ recovered after ROSC during epinephrine + ECMO ⇒ supports anaphylaxis
Time course	Resolves over 60–120 min as block recedes	Persists/worsens until specifically treated	Progressed to cardiac arrest within 17 min of remimazolam infusion onset ⇒ favors anaphylaxis

## Data Availability

All data supporting this case report are presented within the article. Additional clinical details are available from the corresponding author upon reasonable request, subject to institutional and patient privacy protections.

## Supplementary Materials

The following supporting information can be downloaded at: https://www.mdpi.com/article/10.3390/jcm15114099/s1, Table S1: CARE Checklist of information to include when writing a case report.

## Abbreviations

The following abbreviations are used in this manuscript: ABGA, arterial blood gas analysis; ACE, angiotensin-converting enzyme; ACLS, advanced cardiac life support; ARB, angiotensin receptor blocker; ASA, American Society of Anesthesiologists; AV, atrioventricular; BMI, body mass index; BP, blood pressure; CAG, coronary angiography; CARE, CAse REport (reporting guideline); CPR, cardiopulmonary resuscitation; CSF, cerebrospinal fluid; CTPA, computed tomography pulmonary angiography; CVC, central venous catheter; DM, diabetes mellitus; EAACI, European Academy of Allergy and Clinical Immunology; ECMO, extracorporeal membrane oxygenation; eCPR, extracorporeal cardiopulmonary resuscitation; EF, ejection fraction; EKG, electrocardiography; ETT, endotracheal tube; FAERS, FDA Adverse Event Reporting System; GA, general anesthesia; HR, heart rate; HTN, hypertension; IDT, intradermal test; IgE, immunoglobulin E; IJV, internal jugular vein; IV, intravenous; JADER, Japanese Adverse Drug Event Report; JBI, Joanna Briggs Institute; LAE, left atrial enlargement; LVH, left ventricular hypertrophy; MEDLINE, Medical Literature Analysis and Retrieval System Online; NIBP, noninvasive blood pressure; NMBA, neuromuscular blocking agent; OR, operating room; ORi, oxygen reserve index; PE, pulmonary embolism; PEA, pulseless electrical activity; PFT, pulmonary function test; PRISMA, Preferred Reporting Items for Systematic Reviews and Meta-Analyses; PVi, pulse variability index; ROSC, return of spontaneous circulation; RWMA, regional wall motion abnormality; SBP, systolic blood pressure; SpHb, continuous hemoglobin; SpO_2_, peripheral oxygen saturation; SPT, skin prick test; TEE, transesophageal echocardiography; TKR, total knee replacement; TTE, transthoracic echocardiography; V.fib, ventricular fibrillation; WAO, World Allergy Organization.
