# Supplementary material for: Remimazolam-Induced Anaphylaxis After Spinal Anesthesia: A Case Report and Literature Review
Source: J Clin Med. 2026 May 26;15(11):4099. doi: 10.3390/jcm15114099 (PMC13257691; doi:10.3390/jcm15114099)
Supplement: Supplementary file 1 [file jcm-15-04099-s001.zip › jcm-4306716-supplementary.pdf]

CARE Checklist of information to include when writing a case report

Remimazolam-Induced Anaphylaxis After Spinal Anesthesia: A Case Report and Literature Review

Topic	Item	Checklist item description	Reported on page
**Title**	**1**	The words "case report" should be in the title along with what is of greatest interest in this case	**1**
**Key Words**	**2**	The key elements of this case in 2 to 5 key words	**2**
**Abstract**	**3a**	Introduction—What is unique about this case? What does it add to the medical literature?	**1**
	**3b**	The main symptoms of the patient and the important clinical findings	**1**
	**3c**	The main diagnoses, therapeutics interventions, and outcomes	**1**
	**3d**	Conclusion—What are the main "take-away" lessons from this case?	**1–2**
**Introduction**	**4**	Brief background summary of this case referencing the relevant medical literature	**2**
**Patient Information**	**5a**	Demographic information (such as age, gender, ethnicity, occupation)	**5**
	**5b**	Main symptoms of the patient (his or her chief complaints)	**5–6**
	**5c**	Medical, family, and psychosocial history including co-morbidities, and relevant genetic information	**5**
	**5d**	Relevant past interventions and their outcomes	**5**
**Clinical Findings**	**6**	Describe the relevant physical examination (PE) findings	**5–6**
**Timeline**	**7**	Depict important milestones related to your diagnoses and interventions (table or figure)	**5–7**
**Diagnostic Assessment**	**8a**	Diagnostic methods (such as PE, laboratory testing, imaging, questionnaires)	**6, 8–9**
	**8b**	Diagnostic challenges (such as financial, language, or cultural)	**8, 16**
	**8c**	Diagnostic reasoning including other diagnoses considered	**11, 14–15**
	**8d**	Prognostic characteristics (such as staging in oncology) where applicable	**8**
**Therapeutic Intervention**	**9a**	Types of intervention (such as pharmacologic, surgical, preventive, self-care)	**6**
	**9b**	Administration of intervention (such as dosage, strength, duration)	**6**
	**9c**	Changes in intervention (with rationale)	**6**
**Follow-up and Outcomes**	**10a**	Clinician-assessed outcomes and when appropriate patient-assessed outcomes	**8**
	**10b**	Important follow-up test results	**8–9**
	**10c**	Intervention adherence and tolerability (How was this assessed?)	**8**
	**10d**	Adverse and unanticipated events	**6, 8**
**Discussion**	**11a**	Discussion of the strengths and limitations in the management of this case	**15–16**
	**11b**	Discussion of the relevant medical literature	**9–15**
	**11c**	The rationale for conclusions (including assessment of possible causes)	**15–16**
	**11d**	The main "take-away" lessons of this case report	**16**
**Patient Perspective**	**12**	Did the patient share his or her perspective or experience? (Include when appropriate)	**N/A**
**Informed Consent**	**13**	Did the patient give informed consent? Please provide if requested	**Yes ✓ No**
